# Study of phytochemical, anti-microbial, anti-oxidant, and anti-cancer properties of *Allium wallichii*

**DOI:** 10.1186/s12906-017-1622-6

**Published:** 2017-02-08

**Authors:** Jaya Bhandari, BushraTaj Muhammad, Pratiksha Thapa, Bhupal Govinda Shrestha

**Affiliations:** 1 Department of Biotechnology, Kathmandu University, Kathmandu, Nepal; 2 Panjwani Centre for Molecular Medicine and Drug Research, International Centre for Chemical and Biological Sciences, University of Karachi, Karachi, Pakistan

**Keywords:** *Allium wallichii*, FACS, MTT assay, Cancer cell lines, Cytotoxicity, Anticancer agent

## Abstract

**Background:**

There is growing interest in the use of plants for the treatment and prevention of cancer. Medicinal plants are currently being evaluated as source of promising anticancer agents. In this paper, we have investigated the anticancer potential of plant *Allium wallichii*, a plant native to Nepal and growing at elevations of 2300–4800 m. This is the first study of its kind for the plant mentioned.

**Methods:**

The dried plant was extracted in aqueous ethanol. Phytochemical screening, anti-microbial assay, anti-oxidant assay, cytotoxicity assay and the flow-cytometric analysis were done for analyzing different phytochemicals present, anti-microbial activity, anti-oxidant activity and anti-cancer properties of *Allium wallichii*.

**Results:**

We observed the presence of steroids, terpenoids, flavonoids, reducing sugars and glycosides in the plant extract and the plant showed moderate anti-microbial and anti-oxidant activity. The IC_50_ values of *Allium wallichii* in different cancer cell lines are 69.69 μg/ml for Prostate cancer (PC3) cell line, 55.29 μg/ml for Breast Cancer (MCF-7) cell line and 46.51 μg/ml for cervical cancer (HeLa) cell line as compared to Doxorubicin (0.85 μg/ml). The cell viability assay using FACS showed that the IC_50_value of *Allium wallichii* for Burkitt’s lymphoma (B-Lymphoma) cell line was 3.817 ± 1.99 mg/ml.

**Conclusions:**

*Allium wallichii* can be an important candidate to be used as an anticancer agent. Separation of pure compounds with bioassay guided extraction, spectrometric analysis and subsequent cytotoxicity assay of the pure bioactive compounds from *Allium wallichii* is highly recommended as the crude extract itself showed promising cytotoxicity.

## Background

Medicinal plants are considered as potential source for drug development and many novel products have reached clinical trials. Scientists are investigating properties of medicinal plants in order to develop novel drugs against disease like cancer, from natural products. Medicinal herbs have profound scope and have been used to find potential anticancer compounds in them [1]. Use of phytochemicals from the medicinal plants in cancer treatment, may reduce adverse side effects and help to treat cancer and they have in recent years, shown promising anticancer efficiency against different cancers like human mouth epidermal carcinoma (KB cell line), murine leukemia (P388 cell line), human colorectal cancer (BE cell line) and prostate cancer (PC3 cell line) by various mechanisms like up-regulation of p16INK4A, preventing inhibition of MRCK-kinase targeting multiple gene products and targeting mitotic processes [2–8]. Many medicinal plants found in Nepal are also found to have cytotoxic effects against different cancer cell lines. For example, Cell viability assay of plant extracts from *Berberis aristata*; showed a significant cytotoxicity to MDA-MB-231 and U-87 MG human cancer cell line; Withanone from *Withania somnifera* (Ashwagandha) has been identified to have p53-activating tumor-inhibiting property. Ashwagandha leaf powder was non-toxic and anti-tumorigenic in mice assays and caused an abrogation of mortalin-p53 interactions and reactivation of p53 function and it is also established that the different extracts of plant *Juniperus recurva* possess anticancer properties against Breast Cancer (MCF7) cells [9–12].

### *Allium wallichii*(AW)

*Allium wallichii* is a monocotyledonous plant falling in order *Asparagales,* family *Amaryllidaceae and Allioideae* subfamily. It has been used as traditional medicine and spice in Nepal. Recent research papers deal with the genetic diversity and relationship of two species of *Allium* by amplified fragment length polymorphism analysis [13]. The phylogenetic and biogeographic investigation of *Allium sp.* based on nuclear ribosomal internal transcribed spacer and chloroplast ribosomal protein S16 (rps16) sequences implied that genus *Allium* comprises more than 800 species, which defends its place among the largest genera in monocotyledons [14]. Use of plant resource and traditional medicine in Nepal has also been documented for millennia and they are still the most significant health care source for bulk of population [15]. There have been reports of trnK-gene-based molecular phylogenetic studies of plants of genus *Allium* [16]. 1, 2 bis (methylthio) ethene, 2, 4 di-Methiophene, di-Methyl disulfide and di-Methyl trisulfide were established to be the most important volatile constituents of *Allium wallichii* [17]. Diosgenin and Tigogenin are reported as steroidal sapogenins from bulbs of *Allium wallichii.* Kunth, that shows encouraging pharmacological potentials [18]. Neuropsychopharmacological utilization has also been conferred in the studies of the action of *Allium sp.* which shed light to its diverse use [19]. Extracts that includes *Allium sp*. is found to exhibit analgesic and fever relieving effects and can be used for the treatment of inflammation, ulcer, viral infection, cancer, eczema, diabetes, senile gangrene, herpes zoster and most importantly Acquired Immune Deficiency Syndrome (AIDS) [20].

In this paper we have investigated the presence of different phytochemicals in *Allium wallichii* and also established the anti -microbial, anti-oxidant activity of the crude extract of the plant. More importantly this paper is focused in establishing the anticancer potential of the extracts of *Allium wallichii* by *in-vitro* cytotoxicity assay against Prostrate, Cervical and Breast cancer cell lines and Flow-cytometric analysis for viability assay against B-lymphoma cell lines. This is the first study of its kind for the plant *Allium wallichii.*

## Methods

### Collection of plant material

*Allium wallichii* (AW) whole plants were collected from Kathmandu University campus area of Kavrepalanchowk district, Nepal. The plant material was identified by Dr. Rajendra Gyawali, Botanist, Assistant Professor of Department of Pharmacy, Kathmandu University, Nepal. A voucher herbarium specimen is deposited at the herbarium of the Department of Pharmacy, Kathmandu University, Nepal and the voucher specimen number is DoP-H-211. The plant samples were then dried in shade, left over for 15 days and macerated and powdered with the help of kitchen grinder.

### Preparation of plant extract

The dry powder of *Allium wallichii* (100 g) was dipped in 80% Aq. Ethanol for three days stirring twice a day. The solution at the end of third day was filtered and then concentrated in high vacuum-rotary evaporator and finally all the solvent was evaporated using freeze dryer to get the semi-solid plant extract. The same procedure was repeated three times and the freeze-dried extract was mixed together. The extract was then kept in glass vial with airtight caps and stored at 4 °C.

### Phytochemical screening

The phytochemical screening was done using the standard protocols [21–28]. Test for Alkaloids: 5 ml of extract was concentrated to yield a residue. Residue was dissolved in 3ml of 2% (v/v) HCl, few drops of Mayer’s reagent was added. Appearance of the dull white precipitate indicated the presence of basic alkaloids. Test for Coumarin: 4 ml extract solution was taken; 1–2 drops of water (hot) was added. Volume was made half (UV fluorescence). 10% NH_4_OH was added to another half volume (strong fluorescence). Presence of green fluorescence indicated the presence of Coumarin. Test for Saponins: 2 ml extract was shaken vigorously for 30 s in a test tube. Persistence of thick forth even after 30 mins indicated the presence of saponins. Test for Glycosides: 2 ml of extract was dried till 1 ml.1-2 ml NH_4_OH was added and shaken. Appearance of cherish red color indicated the presence of glycosides. Test for Reducing Sugars: 0.5 ml of extract was taken and 1ml distilled water was added. 5-8 drops of Fehling’s solution (hot) was added. Presence of brick red precipitation indicated the presence of reducing sugar. Test for steroids: 1 ml extract was dissolved in 10 ml chloroform. Equal volume of conc. H_2_SO_4_ was added by the side of test tube. Upper layer turned red and sulphuric acid layer turned yellow with green fluorescence. This indicated the presence of steroids. Test for Quinone: 1 ml of extract was taken.1 ml of conc. H_2_SO_4_ was added. Formation of red color indicated the presence of quinone. Test for Terpenoids: 5 ml of extract was taken and mixed with 2 ml of chloroform. 3 ml of conc. H_2_SO_4_ was added to form a layer. Reddish brown precipitate formation at the interface formed indicated the presence of terpenoids. Test for Tannins: About 0.5 g of the dried powdered samples was boiled in 20 ml of water in a test tube and then filtered. Few drops of 0.1% Ferric Chloride was added and observed for brownish green or a blue-black coloration. Test for Flavonoids: A portion of the powdered plant sample was heated with 10 ml of Ethyl Acetate over a steam bath for 3 min. The mixture was filtered and 4 ml of the filtrate was shaken with 1 ml of dilute Ammonia solution. A yellow coloration was observed indicating a positive test for flavonoids.

### DPPH free radical scavenging assay

DPPH (2, 2-diphenyl-1-picrylhydrazyl radical), is a dark-colored crystalline powder composed of stable free-radical molecules. DPPH has major application in laboratory research most notably in anti-oxidant assays. The DPPH assay is typically run by the following standard procedure [29–31]. The hydrogen atom or electron donating abilities of the corresponding extracts/fractions and standards were measured from the bleaching of the purple-colored methanol solution of DPPH. 10 mg DPPH was dissolved in 100 ml methanol (MeOH) to obtain a concentration of 100 μg/ml. The stock solution of the fractions/extracts was prepared by dissolving 25 mg in 50 ml MeOH. Dilutions of the stock solutions of the crude extracts were prepared to obtain concentration of 1 μg/ml, 2 μg/ml, 3 μg/ml, 4 μg/ml, 5 μg/ml and 10 μg/ml while the control was prepared by dissolving 1 ml DPPH in 4 ml MeOH (without sample). 1 ml DPPH was added to each solution and the solution were kept in dark and allowed to stand for exact 30 mins. The UV absorption of each solution was recorded at 517 nm. The reaction was allowed to progress for 30 mins at 37 °C and absorbance was monitored by microplate reader, SpectraMax340 at 517 nm. Upon reduction, the color of the solution fades (violet to pale yellow). Percent Radical Scavenging Activity (% RSA) was determined by comparison with a DMSO containing control. The concentration that causes a decrease in the initial DPPH concentration by 50% is defined as IC_50_ value. Scavenging of free radicals by DPPH as percent radical scavenging activities was calculated as follows. % RSA = Control absorbance – extract absorbance X 100/control absorbance. The IC_50_ values of compounds were calculated by using the EZ-Fit Enzyme kinetics software program (Perrella Scientific Inc. Amherst, MA, USA). Ascorbic acid was used as the reference compound.

### Agar plate diffusion method for anti-microbial activity

Medium was dissolved and autoclaved at 121 °C for 15 min, cooled up to 45 °C and then 40-50 ml media was poured in sterile 14 cm diameter Petri plate, and then allowed to solidify and kept at room temperature as stated in the protocols [32–34].

### Preparation of plant extract

Stock solution of 400 mg/ml was prepared by weighing 200 mg of plant extract in 1.5 ml Eppendorf tube and 0.5 ml of DMSO was added by micropipette. Extract was completely dissolved by vortexing for 5–10 min. Test solution of 200 mg/ml, 100 mg/ml, 50 mg/ml and 25 mg/ml concentrations was prepared.

### Preparation of inoculums

Each culture to be tested was streaked onto nutrient agar plate to obtain isolated colonies. Overnight incubation was done at 37 °C. Then isolated colonies were transferred by the help of sterile loop onto Muller Hinton Broth. Overnight incubation was done at rotary shaker at 37 °C.

### Inoculation

For inoculation, swabbing was done with the help of cotton. Sterilized filter paper discs were dipped into the desirable concentration of the plant extracts and then applied to the plates; incubated at 37 °C for 24 h. After incubation, the diameter of the Zone of inhibition was measured.

### MTT/cytotoxicity assay against cancer cell lines

Cytotoxic activity of plant extract was evaluated in 96-well flat-bottomed micro-titer plates by using the standard MTT (3-[4, 5-dimethylthiazole-2-yl]-2, 5-diphenyl-tetrazolium bromide) colorimetric assay [35]. For this purpose, MCF-7, PC3, HeLa cells were separately cultured in Dulbecco’s Modified Eagle Medium (Sigma Aldrich, Germany), supplemented with 5% of fetal bovine serum (FBS) (Sigma Aldrich, Germany), 100 IU/ml of Penicillin and 100 μg/ml of Streptomycin in 75 cm^2^ flasks and kept in 5% CO_2_ incubator at 37 °C. Exponentially growing cells were harvested, counted with hemocytometer and diluted with a particular medium. Cell culture with the concentration of 1x10^5^ cells/ml was prepared and introduced (100 μL/well) into 96-well plates. After overnight incubation, medium was removed and 200 μL of fresh medium was added with different concentrations of plant extracts. The stock solution of the extract was first prepared (50 μg/ml) in DMSO and was serially diluted up to the concentration of 0.78125 μg/ml. After 48 h, 200 μL MTT (0.5 mg/ml) was added to each well and incubated further for 4 h. Subsequently, 100 μL of DMSO was added to each well. The extent of MTT reduction to formazan within cells was calculated by measuring the absorbance at 570 nm, using a micro plate reader (Spectra Max plus, Molecular Devices, CA, USA). The cytotoxicity was recorded as concentration causing 50% growth inhibition (IC_50_) for the cell lines used. The percent inhibition was calculated by using the following formula: % Inhibition = 100-[(mean of O.D of test compound – mean of O.D of negative control)/(mean of O.D of positive control – mean of O.D of negative control) X 100]. The results (% Inhibition) were processed by using Soft-Max Pro software (Molecular Device, USA). IC_50_ was calculated using EZ-Fit Software.

### Cell viability analysis using PI-On flow cytometry

B-lymphoma cancer cell line was a generous gift from Switzerland, Raji (Burkitt’s lymphoma) (ATCC Catalog. Number: CCl-86). The cell line was maintained at 37 °C in a humidified 5% CO_2_ environment in Roswell Park Memorial Institute (RPMI) 1640 (Caisson, Oklahoma) with 1% L-Glutamine, 1% Penicillin/Streptomycin (Invitrogen, Germany), supplemented with 10% fetal bovine serum (FBS) (PAA laboratories, Austria). The cell viability analysis using PI-On Flow cytometry was performed at Panjwani Centre for Molecular Medicine and Drug Research, International Centre for Chemical and Biological Sciences, University of Karachi, Pakistan using standard protocol [36]. Cell cytotoxicity assay was performed using florescent dye Propidium iodide (Excitation wavelength = 536 nm) (Emission wavelength = 617 nm). The dye has the property that it can’t cross the intact plasma membrane and as a result of this, live cells cannot be stained using this dye. However, the dead cells in which cell membrane integrity is lost, the dye penetrate inside the cell and thus intercalate the DNA of the cell as a result of that only dead cells will fluoresce and could be read in FL2 or FL3 channels/filters in flow-cytometer. In order to generate dose response curve of standard and test compounds against the cell line, cells were seeded in round bottom 96 well plate in such a way that each well contain 1.3x10^5^ cells per well with 200 μl final reaction volume. Negative control wells were given media containing cells only while in positive control wells 0.5% of the total reaction volume of DMSO was added.

## Results

The yield percentage of plant extract from the cold extraction was calculated. Yield percentage was calculated by using following formula: Yield = (Weight of the extract obtained)/(Total weight of the sample loaded) x 100%

$$ Yield\%=\frac{12.07}{100}\times 100\%=12.07\% $$

In the phytochemical screening, as shown in Table 1, we observed the presence of different phytochemicals like steroids, terpenoids, flavonoids, reducing sugars and glycosides. Anti-microbial assay was performed with the plant extract of *Allium wallichii* by agar plate diffusion method. Five different antibiotics were used as standard drugs. Eight different microorganism *B. cereus, B. thuringiensis, E.coli, P. aeruginosa, P. mirabilis, Rhizopus, A. flavus and B. subtilis* were used for the assay.

**Table 1:** Results of phytochemical screening of *Allium wallichii*

Phytochemicals	*Allium wallichii*
Alkaloids	-
Tannins	-
Flavonoids	+
Reducing Sugars	+
Coumarin	-
Glycosides	+
Quinone	-
Steroids	+
Terpenoids	+
Saponins	-

The anti-microbial activity was assayed by measuring the Zone of inhibition (ZOI) of different extracts on the agar disc plate. The anti-microbial test of the *Allium wallichii* was done in different concentrations of the plant used so as to study the concentration dependent toxicity against the different bacteria and fungi. The different concentrations used such were 200, 100, 50 and 25 mg/ml. The standard antibiotics used were Gentamicin (GEN10), Ciprofloxacin (CF30), Chloramphenicol (C30), Cephotaxime (CTX30) and Tetracycline (TE30) as presented in Table 2. The highest ZOI among the five standard antibiotics used was of CTX 30 against *Rhizopus* as stated in the Table 2.

**Table 2:** Zone of Inhibition shown by standard antibiotics

Zone of Inhibition shown by standard antibiotics								
	*B. cereus*	*E. coli*	*B. thuringiensis*	*P. mirabilis*	*Rhizopus*	*A. flavus*	*P. aerugenosa*	*B. subtilis*
CTX30	20	25	9	20	36	0	29	21
GEN10	27	29	30	30	27	0	15	23
CF30	34	35	35	35	30	0	40	27
C30	30	30	12	7	25	0	23	21
TE30	18	30	20	9	29	0	28	25

**Fig. 1:**
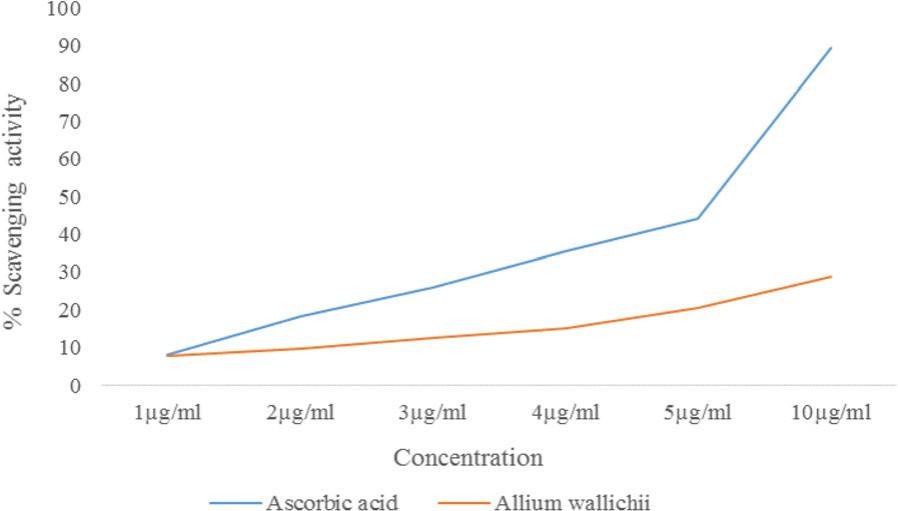
Comparison of Percentage Scavenging of DPPH by *Allium wallichii* at the concentration range (1-10 μg/ml) as compared to Ascorbic acid

The plant showed concentration dependent anti-microbial activity towards bacteria used with the highest ZOI observed being 12 mm for *B.cereus* at the highest concentration used i.e. 200 mg/ml as shown in Table 3. The percentage scavenging activity was measured at different concentrations of the extracts 1 μg/ml, 2 μg/ml, 3 μg/ml, 4 μg/ml, 5 μg/ml and 10 μg/ml. The comparison showed that the percentage scavenging activity is concentration dependent but the line plot of *Allium wallichii* is observed below the line of Ascorbic acid, which corresponds to the moderate activity as compared to the standard Ascorbic acid. The percentage scavenging activity of *Allium wallichii (AW)* was found to be 7.68, 9.79, 12.48, 14.98, 20.62 and 28.66 as compared to 8.07, 18.45, 25.81, 35.46, 44.25 and 89.25 of Standard Ascorbic acid at concentration of 1 μg/ml, 2 μg/ml, 3 μg/ml, 4 μg/ml, 5 μg/ml 10 μg/ml respectively. The IC_50_ value was calculated from the graph of regression analysis and found to be 17.87 μg/ml as shown in Fig. 1.

**Table 3:** Results of antimicrobial assay of *Allium wallichii* against different microorganisms

Plant	Microbes	Extract concentration (mg/ml) and Zone of inhibition (mm)			
		200 mg/ml	100 mg/ml	50 mg/ml	25 mg/ml
*Allium wallichii*	*B. cereus*	12	11	9	9
	*B. thuringiensis*	9	9	8	7
	*E. coli*	7.5	7.5	6.5	6.5
	*P. aeruginosa*	8	8	6	6
	*P. mirabilis*	7.5	7	7	6.5
	*Rhizopus*	7.5	7.5	7	6.5
	*A. flavus*	9	7.5	7	6.5
	*B.subtilis*	7	7	7	6.5

**Fig. 2:**
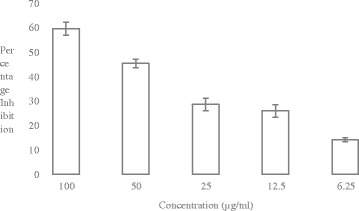
Percentage Inhibition of *Allium wallichi* against Prostate Cancer (PC3) Cell lines. (*n* = 3, Values are Mean ± SD)

**Fig. 3:**
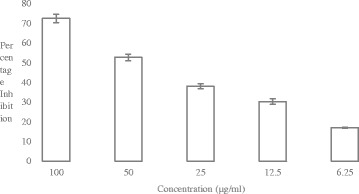
Percentage Inhibition of *Allium wallichi* against Cervical Cancer (HeLa) cell lines. (*n* = 3, Values are Mean ± SD)

Study of cytotoxicity of *Allium wallichii* against Prostate Cancer (PC3) Fig. 2 and Cervical Cancer (HeLa) Fig. 3 cell lines revealed that the plant studied was moderately cytotoxic to both the cancer cell lines. The cytotoxic effect of the plant was compared with the standard (Doxorubicin). *Allium wallichii* showed 59.48 ± 2.58%, 45.32 ± 1.82%, 28.53 ± 2.59%, 22.58 ± 4.91% and 10.70 ± 5.66 of percent cytotoxicity at 100 μg/ml, 50 μg/ml, 25 μg/ml, and 12.5 μg/ml and 6.25 μg/ml concentration of the extracts, respectively, against PC3 cell lines. The IC_50_ value of *Allium wallichii* was 69.69 μg/ml for PC3. Similarly, the percent cytotoxicity for HeLa cell lines was 72.45 ± 2.12%, 52.61 ± 1.66%, 37.88 ± 1.26%, 30.24 ± 1.33% and 16.92 ± 0.27% respectively at the above mentioned concentration with calculated IC_50_ value of 46.51 μg/ml as compared to Doxorubicin (0.85 μg/ml). All IC_50_ values of the extracts were calculated using EZ-Fit Software. From these findings, moderate cytotoxic activity of *Allium wallichii* was observed towards different cancer cell lines taken for study. We observed moderate cytotoxicity of *Allium wallichii* crude extracts against Breast Cancer (MCF-7) cell lines (Fig not shown) with the percentage inhibition of 45.22% at 50 μg/ml concentration with the calculated IC_50_ value of 55.29 μg/ml.

**Fig. 4:**
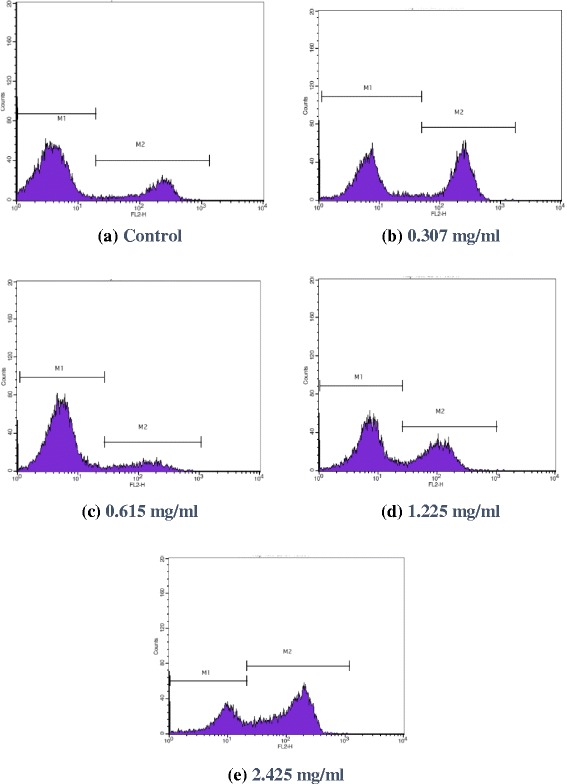
The Histogram plot of the viable and dead cell count from the flow cytometric analysis for Burkitt’s lymphoma cell line. Fig (**a**) Control, Fig (**b**-**e**) *Allium wallichi* at different concentrations

Flow cytometry analysis results, as seen in Fig. 4 showed that, marker M1 is placed around the events that are negative to florescence label, and Marker M2 is placed to the right of M1 to designate positive events. In our work since Propidium Iodide dye (detected at FL2 and FL3 both) is used, which holds the capability to enter into cells with compromised cell membrane (dead cells) consequently dead cells will take up the dye and will show in high florescent region M2 (left part) and viable cells will be in M1 (right half of the histogram). The Fig. 4, shows the viability assay for the total events gated by gate R1 of control and extracts at different concentrations against Burkitt’s lymphoma cell lines. As shown in Fig. 4, for control (a) M1 corresponds to viable 75.45% and M2 correspond to dead 21.01%. For the *Allium wallichii* at 0.31 mg/ml concentration (b) shows 53% viable and 46.10% dead cells. With further increasing concentrations to 0.62 mg/ml (c) 85% viable and 13.37% dead cells were observed. Similarly, at 1.23 mg/ml (d) 60.70% viable and 38.73% dead cells and at 2.43 mg/ml (e) 32% viable and 66.98% dead cells were observed. Analyzing all the above data, the IC_50_ value for *Allium Wallichii* against Lymphoma cells used in the flow-cytometric analysis line is found to be 3.817 ± 1.99 mg/ml which demonstrate good cytotoxicity against Lymphoma cell lines.

## Discussions

The yield percentage of 12.07%, for the plant shows that it has various compounds in it that could have health benefits. Phytochemicals like steroids, terpenoids, flavonoids and glycosides have been shown to have anti-cancer activity. For the anti-microbial assay, the gradual decrease in concentration showed corresponding decrease in ZOI. The microorganism like *E. coli, P. mirabilis* and *Rhizopus* were found to be less susceptible toward the action of *Allium wallichii.* We can infer from the comparison between the data from Table 2 and Table 3 that, the studied plant show only moderate antimicrobial activity. This can be attributed to the fact that the whole plant including the tuber of *Allium wallichii* along with leaves has been used for the study; and there must be significant difference in the phytochemicals present.

The free radical species can start chain reactions and can cause damage or death to the cell. Antioxidants terminate these chain reactions by removing free radical intermediates, and inhibit other oxidation reactions. Antioxidants compounds scavenge free radicals such as peroxide, hydrogen peroxide or lipid peroxyl and thus inhibit the oxidative mechanisms that lead to degenerative diseases. Screening of plants for this is done by measuring the antioxidant activity by various in vitro activities such as DPPH method, Nitric Oxide method and in vivo models using rats and mice. Free radicals that are commonly used to assess antioxidant activity in vitro is 2, 2-diphenyl-1-picrylhydrazyl (DPPH). So higher the percentage scavenging activity, higher the antioxidant activity and higher the anticancer property it may possess. The results in Fig. 1 show that, the plant have moderate anti-oxidant activity.

The extract of the plant showed moderate anti-cancer activity, when studied with cancer cell lines namely PC3, HeLa and MCF-7. The calculated IC_50_ of the plant shows that the plant can have anti-cancer activity as has been reported for other plants viz. *Caralluma adscendens (Roxb.)*, *Daedalea gibbosa, Alpinia conchigera, Chelidonium majus*, *Ocimum sanctum Linn, Potentilla erecta*, *Chamaenerium angustfolium*, *Paphia undulata, Filipendula ulmaria*, *Inula helenium* against different cell lines [36–42]. Analyzing the data of FACS, the IC_50_ value for *Allium Wallichii* against the Lymphoma cell used demonstrate good cytotoxicity. The moderate cytotoxicity in the studies might have been due to use of whole plant for the assay. The separation of pure compounds with bioassay guided extraction and the spectrometric analysis and subsequent cytotoxicity check would reveal the bioactive components and give a better activity. Thus, most of the research these days are directed towards separation of bioactive components.

## Conclusions

From the experiments performed, we concluded the presence of different phytochemicals like steroids, terpenoids, flavonoids, reducing sugars and glycosides in the plant *Allium wallichii*. In the DPPH-RSA assay *Allium wallichii* showed moderate antioxidant activity; while anti-microbial assay also showed moderate activity against known pathogens. Further observations demonstrated that the plant *Allium wallichii* exhibits moderate cytotoxic activity towards Prostate cancer (PC3), cervical cancer (HeLa) and Breast Cancer (MCF-7) cell lines. Finally, the flow-cytometric analysis demonstrated a good cytotoxicity of *Allium wallichii* against Lymphoma cell lines with significant cell death in the population of the Lymphoma cell line used. These results accounts for the fact that, *Allium wallichii,* together with moderate anti-oxidant and cytotoxic activity and presence of important phytochemicals like flavonoids, steroids, glycosides and terpenoids, can be a very important candidate to be used as an anticancer agent. Moreover, use of plant parts separately and the isolation of pure compounds from the plant would certainly have better activity against cancer.

## Abbreviations

C30: Chloramphenicol
CF30: Ciprofloxacin
CTX30: Cephotaxime
GEN10: Gentamicin
TE30: Tetracycline
ATCC: American type culture collection
DMSO: Dimethyl sulfoxide
DPPH: 2,2-diphenyl-1-picrylhydrazyl
FACS: Fluorescence-activated cell sorting
FBS: Fetal bovine serum
FL2 or FL3: Channels/filters in flow cytometer
FSC-H: Forward scatter in flow cytometry; H refers to height
HeLa: Cervical cancer cell line
IC_50_: Half maximal inhibitory concentration
MCF-7: Human breast cancer cell line
MDA-MB-231: Human mammary cancer cell line
MTT-: 3-(4,5-dimethylthiazol-2-yl)-2,5-diphenyltetrazolium bromide
OD: Optical density
p53: Tumor suppressor gene p53
PC3: Human prostate cancer cell line
RPMI: Roswell park memorial institute
SSC-H: Sidewise scatter in flow cytometry; H refers to height
U-87 MG: Human primary glioblastoma cell line
ZOI: Zone of inhibition

## References

[1] Riaz M, Zia-Ul-Haq M, Saad B (2016). Anthocyanins and Human Health: Biomolecular and Therapeutic Aspects. Springer Int Publishing.

[2] Desai AG, Qazi GN, Ganju RK, El-Tamer M, Singh J, Saxena AK et al. Medicinal plants and cancer chemoprevention. Current Drug Metabolism. 2008; doi:10.2174/138920008785821657. 10.2174/138920008785821657 PMC4160808 18781909

[3] Manosroi J, Dhumtanom P, Manosroi A. Anti-proliferative activity of essential oil extracted from Thai medicinal plants on KB and P388 cell lines. Cancer Letters (Amsterdam, Netherlands). 2006; doi:10.1016/j.canlet.2005.04.021. 10.1016/j.canlet.2005.04.021 15979235

[4] Krifa M, Pizzi A, Mousli M, Chekir GL, Leloup L, Ghedira K (2014). *Limoniastrum guyonianum* aqueous gall extract induces apoptosis in colorectal cancer cells by inhibiting calpain activity. Tumor Biol.

[5] Patil SD, Chaudhari MA, Sapkale PV, Chaudhari RB (2013). A recent review on anticancer herbal drugs. J Drug Discov Ther.

[6] Sung B, Prasad S, Yadav VR. Aggarwal BB. Cancer Cell Signaling Pathways Targeted by Spice-Derived Nutraceuticals. Nutrition and Cancer. 2012; doi:10.1080/01635581.2012.630551. 10.1080/01635581.2012.630551 PMC3645308 22149093

[7] Lowe HC, Watson CT, Badal S, Toyang NJ, Bryant J. Cycloartane-3,24,25-triol inhibits MRCK- kinase and demonstrates promising anti-prostate cancer activity in vitro. Cancer Cell International. 2012; doi:10.1186/1475-2867-12-46. 10.1186/1475-2867-12-46 PMC3520828 23151005

[8] Rao CV, Kurkjian CD, Yamada HY (2012). Mitosis-targeting natural products for cancer prevention and therapy. Curr Drug Targets.

[9] Lamichhane B, Adhikari S, Shrestha P, Shrestha BG. Study of phytochemical, antioxidant, anti-microbial and anticancer activity of Berberis aristata. The Journal of Tropical Life; Science Open Access. 2014;4(1):1–7.

[10] Widodo N, Kaur K, Shrestha BG, Nagpal A, Takagi Y, Kaul SC (2007). Selective Killing of Cancer Cells by Leaf Extract of Ashwagandha: Identification of a Tumor Inhibitory Factor and The First Molecular Insights to its Effect. Clin Cancer Res.

[11] Widodo N, Takagi Y, Shrestha BG, Tetsuro I, Kaul SC, Wadhwa R (2008). Selective killing of cancer cells by leaf extract of Ashwagandha: Components, activity and pathway analyses. Cancer Lett.

[12] Bhandari J, Niraula P, Thapa P, Thapa N, Shrestha N, Shrestha BG. Phytochemical screening, antioxidant assay of Juniperus recurva and study of it’s in vitro cytotoxicity against breast cancer cell lines. Int J Pharma Bio Sciences. 2015;6(3) (B):1134-45.

[13] Wang J, He CZ, Dang CL, Huang RF (2011). Genetic diversity and relationship of *Allium tchongchanense* and *A. wallichii* based on AFLP analysis. Guihaia.

[14] Li QQ, Zhou SD, He XJ, Zhang YC, Wei XQ. Phylogeny and biogeography of *Allium* (*Amaryllidaceae: Allieae*) based on nuclear ribosomal internal transcribed spacer and chloroplast rps 16 sequences, focusing on the inclusion of species endemic to China. Ann Botany. 2010; doi:10.1093/aob/mcq177. 10.1093/aob/mcq177 PMC2958792 20966186

[15] Kunwar RM, Nepal BK, Kshhetri HB, Rai SK, Bussmann RW. Ethnomedicine in Himalaya: a case study from Dolpa, Humla, Jumla and Mustang districts of Nepal. Journal of ethnobiology and ethnomedicine. 2006; doi:10.1186/1746-4269-2-27. 10.1186/1746-4269-2-27 PMC1513199 16749924

[16] Zhou SD, He XJ, Ge S (2006). trnK-gene-based molecular phylogeny of *Allium* plants(*Lilaceae)*. Acta Bot Boreal-Occidenta Sin.

[17] Kattel A, Maga JA (1995). Volatile compounds from dried Jimbu (*Allium wallichii*). Dev Food Sci.

[18] Kamal R, Sharma GL (1984). Steroidal sapogenins from bulbs of *Allium wallichii* Kunth. Pharmazie.

[19] Dua JS, Prasad DN, Tripathi AC, Gupta R (2009). Role of traditional medicine in neuropsychopharmacology. Asian J Pharm Clin Res.

[20] Meier. The process of the herbal drug to the herbal medicinal product. Therapeutische Umschau. 2002;59(6):275–82. 10.1024/0040-5930.59.6.275 12125176

[21] Uddin GA, Rauf M, Arfan M, Ali M, Qaisar M (2012). Saadiq, Atif M. Preliminary phytochemical Screening and antioxidant activity of Bergenia Ciliata. Middle-East J. Sci Res.

[22] Uddin G, Rauf A, Qaisar M, Latif A, Ali M (2011). Preliminary phytochemical screening and antimicrobial activity of hedera helix L. Middle-East J Sci Res.

[23] Nisar M, Ali S, Qaisar M (2011). Preliminary Phytochemical Screening of Flowers, Leaves, Bark, Stem and Roots of Rhododendron arboretum. Middle-East J Sci Res.

[24] Philip D, Kaleena PK, Valivittan K, Girish Kumar CP (2011). Phytochemical Screening and Antimicrobial Activity of Sansevieria roxburghiana Schult. and Schult. F. Middle-East J Sci Res.

[25] Raju DC, Victoria TD, Biji N, Nikitha G (2015). Evaluation of Antioxidant Potential of Ethanolic Extract of Centella asiatica L. Res J Pharm Technol.

[26] Uddin G, Rauf A (2012). In vitro Antimicrobial Profile of Pistacia integerrima Galls Stewart. Middle-East J Med Plants Res.

[27] Usman R, Khan A, Gul S, Rauf A, Muhammad N (2012). Evaluation of In vitro Anti-Oxidant properties of Selected Medicinal Plants. Middle-East J Med Plants Res.

[28] Usman R, Khan A, Gul S, Rauf A, Muhammad N (2012). Preliminary Anti-Oxidant Profile of Selected Medicinal Plants of Pakistan. Middle-East J Med Plants Res.

[29] Thadhani V, Choudhary MI, Ali S, Omar I, Siddique H, Karunaratne V (2011). Antioxidant activity of some lichen metabolites. Nat Prod Res.

[30] Roots R, Okada S (1975). Strand break formation in plasmid DNA irradiated in aqueous solution: effect of medium temperature and hydroxyl radical scavenger concentration. J Radiat Res.

[31] Lee SK, Mbwambo ZH, Chung H, Luyengi L, Gamez EJ, Mehta RG, Kinghorn AD, Pezzuto JM (1998). Evaluation of the antioxidant potential of natural products. Comb Chem High Throughput Screen.

[32] Alves TM, Silva A, Brandao AF, Grandi M, Smania T, Smania Zani CL (2000). Biological screening of Brazilian medicinal plants. Mem del Inst Oswaldo Cruz.

[33] Stepanovic S, Antic N, Dakic I, Svabic Vlahovic M (2003). In vitro antimicrobial activity of propolis and synergism between propolis and antimicrobial drugs. Microbiol Res.

[34] Carron RA, Maran JM, Montero-Fernandozaigo L, Dominguez AA (1987). Synthesis, characterization and biological studies of tri and di organotin(IV) complexes with 2,4 dofluoro-4-hydroxy-[1,1,]-biphenyl-3-carbolic acid: Crystal structure of [(CH 3) Sn(C13H7O3F2)]. Plantes Med Phytother.

[35] Mosmann T (1983). Rapid colorimetric assay for cellular growth and survival: application to proliferation and cytotoxicity assays. J Immunol Methods.

[36] Tung JW, Parks DR, Moore WA, Herzenberg LA, Herzenberg LA (2004). Identification of B cell subsets: an exposition of 11-color (Hi-D) FACS methods. Methods Mol Biol.

[37] Gowri S, Chinnaswamy P (2012). Effect of Carallumaadscendens (Roxb.) on lipid peroxidation and antioxidant status in 1, 2-dimethyl hydrazine induced experimental colon carcinogenesis. J Pharm Res.

[38] Yassin M, Wasser SP, Mahajna J (2008). Substances from the medicinal mushroom Daedalea gibbosa inhibit kinase activity of native and T315I mutated Bcr-Abl. Int J Oncol.

[39] Bhattacharyya P, Bishayee A. Ocimum sanctum Linn. (Tulsi): an ethnomedicinal plant for the prevention and treatment of cancer. Anti-cancer Drugs. 2013; doi:10.1097/CAD.0b013e328361aca1 10.1097/CAD.0b013e328361aca1 23629478

[40] Aun LL, Azmi MN, Ibrahim H, Awang K, Nagoor NH. 1'S-1'-acetoxyeugenol acetate: a novel phenylpropanoid from Alpiniaconchigera enhances the apoptotic effects of paclitaxel in MCF-7 cells through NF-β inactivation. Anticancer Drugs. 2011; doi:10.1097/CAD.0b013e328343cbe6. 10.1097/CAD.0b013e328343cbe6 21346553

[41] Spiridonov NA, Konovalov DA, Arkhipov VV. Cytotoxicity of some Russian ethnomedicinalplants and plant compounds. Phytotherapy Research. 2005; doi:10.1002/ptr.1616. 10.1002/ptr.1616 16106386

[42] Hongmian W, Xiuping F, Xiaoling L, Xueqiong H. Isolation and extraction of glycosaminoglycan from Paphia undulata and the study of its anti-tumor activity. Food and Fermentation Industries. 2005;7:39.

